# The kidney anion exchanger 1 affects tight junction properties via claudin-4

**DOI:** 10.1038/s41598-019-39430-9

**Published:** 2019-02-28

**Authors:** Rawad Lashhab, Alina C. Rumley, Denis Arutyunov, Midhat Rizvi, Charlotte You, Henrik Dimke, Nicolas Touret, Richard Zimmermann, Martin Jung, Xing-Zhen Chen, Todd Alexander, Emmanuelle Cordat

**Affiliations:** 1 Department of Physiology and Membrane Protein Disease Research Group, University of Alberta, Edmonton, Alberta Canada; 2 Cardiovascular and Renal research, University of Southern Denmark, Odense, Denmark; 3 Department of Biochemistry and Membrane Protein Disease Research Group, University of Alberta, Edmonton, Alberta Canada; 4 Department School of Medicine, Medical Biochemistry and Molecular Biology, Saarland University, Homburg, Germany; 5 Department of Nephrology, Odense University Hospital, Odense, Denmark

## Abstract

In the renal collecting duct, intercalated cells regulate acid-base balance by effluxing protons through the v-H^+^-ATPase, and bicarbonate via apical pendrin or the basolateral kidney anion exchanger 1 (kAE1). Additionally, collecting duct cells play an essential role in transepithelial absorption of sodium and chloride. Expression of kAE1 in polarized MDCK I cells was previously shown to decrease trans-epithelial electrical resistance (TEER), suggesting a novel role for kAE1 in paracellular permeability. In our study, we not only confirmed that inducible expression of kAE1 in mIMCD3 cells decreased TEER but we also observed *(i)* increased epithelial absolute permeability to both sodium and chloride, and *(ii)* that this effect was dependent on kAE1 activity. Further, kAE1 regulated tight junction properties through the tight junction protein claudin-4, a protein with which it physically interacts and colocalizes. These findings unveil a novel interaction between the junctional protein claudin-4 and the kidney anion exchanger, which may be relevant to ion and/or pH homeostasis.

## Introduction

Type A intercalated cells in the distal nephron are essential to maintain a balanced plasma pH. These cells secrete protons generated by cytosolic carbonic anhydrase II into the lumen while transporting bicarbonate back into the blood. This physical separation of acids and bases is mediated by the apical v-H^+^-ATPase and the basolateral kidney anion exchanger 1 (kAE1).

kAE1 is a 14 transmembrane segments dimeric glycoprotein with cytosolic amino- (N) and carboxyl (C)-terminal ends^1^. The kAE1 transmembrane domain is sufficient for the exchange of chloride and bicarbonate ions and encompasses the binding site for stilbene derivatives. It also carries the N-glycosylation site at position 642 (numbering as per the erythroid isoform). The N-terminus is truncated by the first 65 amino acids present in the erythroid form of the protein, while a short C-terminus is conserved in both erythroid and renal isoforms^2^. This cytosolic domain interacts with various proteins including carbonic anhydrase II^3^, adaptor protein 1 A&B^4–6^, glyceraldehyde phosphate dehydrogenase^7^, peroxiredoxin 6^8^, and contains a putative type I PDZ binding domain^9^, which interacts with PDLIM5^10^.

Defects in the genes encoding carbonic anhydrase II, the v-H^+^-ATPase or basolateral kAE1 can lead to distal renal tubular acidosis (dRTA)^11^. This disease is characterized by a metabolic acidosis, hypokalemia, hyperchloremia, nephrocalcinosis and renal failure if untreated. Interestingly, Sebastian and colleagues observed that even after sustained correction of the metabolic acidosis, RTA patients fail to conserve sodium and chloride ions^12^. Using MDCK cells as a model for intercalated cells, dRTA originating from mutated SLC4A1 gene that encodes for kAE1 was proposed to arise either from an inactive mutant, from mis-trafficking of this protein to either intracellular compartments, or to the apical membrane^13–18^. However, recent evidence obtained from human biopsies^19^ and mice knocked in with the dominant dRTA mutation R607H (equivalent of the R589H in humans), which developed incomplete dRTA, suggests that the origin of the disease is much more complex than so far anticipated^20^. Indeed, in type-A intercalated cells from homozygous R607H knocked-in mice, the mutated protein was found to be functional and located at the basolateral membrane, while apical v-H^+^-ATPase failed to relocate to the luminal membrane upon acidic conditions, thus giving rise to incomplete dRTA. These recent findings highlight the fact that the molecular and cellular mechanisms leading to dRTA are still poorly understood.

In an effort to decipher how intercalated cells maintain normal plasma pH homeostasis, we focused our efforts on the intriguing and un-explained finding from Toye and colleagues who showed that kAE1 expression in MDCK I cells results in a leaky epithelium to apically applied fluorescently labelled biotin molecules^15^. These findings support that expression of kAE1 somehow affects tight junction permeability. Taking into account this latter report together with the renal loss of sodium and chloride in RTA patients^12^, we hypothesized that defective kAE1 function as seen in dRTA patients results in a tighter collecting duct epithelium, and may result in urinary loss of sodium and chloride.

In this manuscript, we report the characterization of the tight junction properties of mouse inner medullary collecting duct (mIMCD3) cells inducibly expressing kAE1. We provide evidence that the increased leakiness of kAE1-expressing mIMCD3 cells is mediated by an effect on claudin-4, a paracellular pore to chloride ions that is expressed in principal cells and intercalated cells of the collecting duct and which physically interacts with kAE1 protein.

## Results

### kAE1 expression results in decreased transepithelial electrical resistance (TEER)

**Figure 1:**
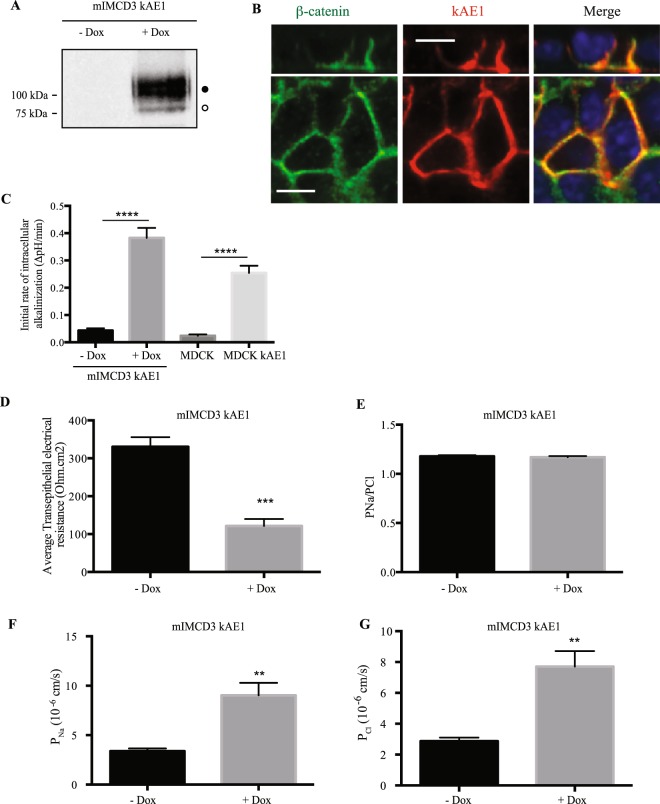
Expression of kAE1 in mIMCD3 cells decreases trans-epithelial electrical resistance (TEER) and increases transepithelial sodium and chloride ion fluxes. (**A**) mIMCD3 cells expressing kAE1 in an inducible manner were incubated with or without doxycycline for 24 hours, prior to lysis and analysis of protein abundance by immunoblot. Mouse anti-HA antibody was used to detect kAE1 protein. Black and white circles correspond to kAE1 carrying complex and high mannose oligosaccharides, respectively. (**B**) mIMCD3 cells expressing kAE1 were grown to full polarization on semi-permeable filters for 10 days and induced with doxycycline for 24 hours prior to immunostaining with either anti-β-catenin antibody (green) or anti-HA antibody (red). The nuclei were stained with DAPI (blue). (**C**) 70% confluent mIMCD3 cells expressing kAE1 were induced with doxycycline for 24 hours prior to loading the cells with BCECF-AM in the presence of NaCl. Upon switching the extracellular solution to one containing Na gluconate instead of NaCl, the initial rate of intracellular alkalinization was recorded for the first 60 seconds and plotted as a function of time (see methods for further details). Error bars correspond to means ± SEM, n = 5 at minimum, ****P < 0.0001 versus “mIMCD3 kAE1 – Dox” or “MDCK” condition using one-way ANOVA. (**D**) Ussing chambers measurements of TEER showing that kAE1 expression results in decreased TEER but unchanged P_Na_/P_Cl_ ratio. (**E**) However, both absolute permeabilities to sodium (**F)** and chloride (**G**) increased upon kAE1 expression. Error bars correspond to means ± SEM, n = 4 at minimum, **P < 0.01 and ***P < 0.001 versus “– Dox” condition using un-paired t-test.

In MDCKI cells, Toye and colleagues reported that stably expressing kAE1 protein resulted in an increased leakiness of the epithelial monolayer to fluorescently labelled biotin when added to the luminal side of the monolayer^15^. To further assess the effect of kAE1 in epithelial properties, we infected mIMCD3 cells with lentiviruses containing kAE1 cDNA whose expression was inducible upon incubation with doxycycline. This strategy also allowed us to avoid the progressive loss of kAE1 expression seen when it is constitutively expressed^18,21^. Upon induction, we observed that kAE1 protein was located at the basolateral membrane, and carried complex oligosaccharides (Fig. 1A,B), supporting a similar processing as in other cell lines and in mouse kidney as previously described^15,18,20^. The protein was functional as we measured the initial rate of intracellular alkalinization of 0.38 ± 0.04 ΔpH/min (n = 9) compared to 0.04 ± 0.01 ΔpH/min (n = 9) in the absence of doxycycline. The initial rate of intracellular alkalinization was significantly higher than that measured in MDCK cells stably expressing kAE1 WT (0.25 ± 0.03 ΔpH/min, n = 5) (Fig. 1C), due to a higher expression level in mIMCD3 cells (data not shown). The data interpretation therefore supports that in this cell line, kAE1 behaves in a similar way to intercalated cells. To assess tight junction properties, cells grown for a minimum of 10 days on semi-permeable Transwell filters were mounted onto Ussing chambers and TEER was measured. While the initial TEER value was 314.5 ± 27.2 ohm*cm^2^ (n = 4) in mIMCD3 cells in the absence of doxycycline, the TEER significantly decreased to 121.4 ± 18.5 ohm*cm^2^ (n = 3) upon cell incubation with doxycycline for 24 hours (Fig. 1D). Of note, this decrease was not due to doxycycline since incubation of non-infected mIMCD3 cells with this chemical did not alter the TEER (Supplemental Figure). This result is consistent with kAE1 expression affecting tight junction properties by reducing the tightness of the epithelium (193 ohm*cm^2^ reduction in the TEER value upon kAE1 expression), and with the former observation from Toye and colleagues^15^.

We next measured the dilution potential generated after the basolateral compartment was diluted to approximately 50% of the concentration of sodium and chloride and we then calculated the absolute permeability to sodium and chloride using the Hodgkin-Katz equation. Although the sodium to chloride absolute permeability ratio remained unchanged upon incubation with doxycycline (P_Na_/P_Cl_ of 1.18 ± 0.01 and 1.17 ± 0.01, n = 3, in the absence and presence of doxycycline, respectively) (Fig. 1E), the absolute permeabilities to both ions increased. The permeability to sodium increased from 3.3 ± 0.3 10^−6^ cm/s to 9.0 ± 0.1 10^−6^ cm/s and that of chloride increased from 2.9 ± 0.2 10^−6^ cm/s to 7.7 ± 0.1 10^−6^ cm/s upon doxycycline incubation (Fig. 1F,G). This result indicates that expression of kAE1 renders the renal epithelium more leaky to both anions and cations.

### The function of kAE1 is essential to alter TEER

**Figure 2:**
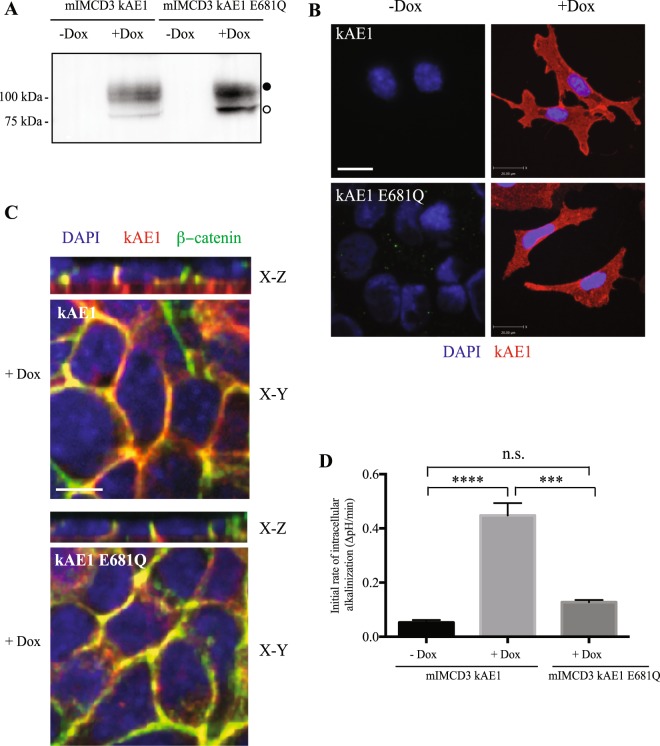
kAE1 E681Q mutant reaches the plasma membrane but is inactive. (**A**) Immunoblot of mIMCD3 cells either expressing kAE1 WT or E681Q mutant. Both proteins are expressed at comparable levels and both carry complex (black circle) and high mannose (white circle) oligosaccharides. (**B**) mIMCD3 cells stably expressing kAE1 WT or E681Q mutant were grown on glass coverslips and incubated with doxycycline to induce kAE1 protein expression. Cells were then immunostained with an anti-HA antibody followed by Cy3 coupled secondary antibody (red). Blue staining corresponds to nuclear localization with DAPI. Bar = 20 μm. (**C**) mIMCD3 cells stably expressing kAE1 WT or E681Q mutant and incubated with doxycycline (+Dox) were grown on semi-permeable filters, fixed, permeabilized and incubated with rabbit anti-β-catenin (green) and mouse anti-HA antibodies (red). Nuclei were stained with DAPI (blue). X-Y shows a middle section through the cells, X-Z shows a side view of the cells. Bar = 10 μm. (**D**) Functional assay using pH-sensitive fluorescent probe BCECF on either mIMCD3 cells stably expressing kAE1 WT without doxycycline (mIMCD3 kAE1 - Dox), with doxycycline (mIMCD3 kAE1 + Dox) or kAE1 E681Q with doxycycline (mIMCD3 kAE1 E681Q + Dox). Error bars correspond to means ± SEM, n = 3. ***P < 0.001 versus “mIMCD3 kAE1 + Dox” condition, ****P < 0.0001 versus “mIMCD3 kAE1 - Dox” condition, n.s. indicates no significant difference with “mIMCD3 kAE1 – Dox” condition using one-way ANOVA.

**Figure 3:**
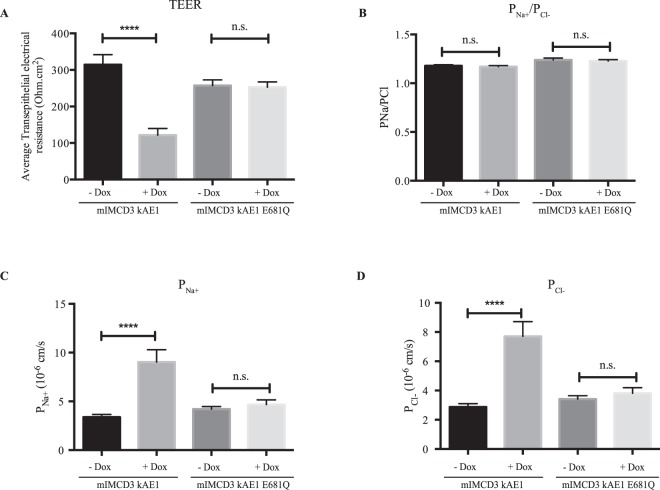
The kAE1 E681Q mutant does not affect tight junction properties. mIMCD3 cells stably expressing kAE1 E681Q mutant were grown on semi-permeable filters for 10 days prior to mounting in Ussing chambers after inducing protein expression (+Dox). Measurements of TEER (**A**), P_Na_/P_Cl_ ratio (**B**) and absolute permeability to sodium (**C**) or chloride (**D**) show that kAE1 E681Q mutant expression does not affect tight junction permeability, compared to kAE1 WT (mIMCD3 kAE1). Error bars correspond to means ± SEM, n = 7. No significant (n.s.) difference was observed in any of the conditions (One-way ANOVA) for mIMCD3 kAE1 E681Q cells, ****P < 0.0001 versus “mIMCD3 kAE1 - Dox” condition.

As erythroid AE1 plays an important scaffolding role in red blood cells^22^, we next wondered whether the decrease in tightness of the monolayer was due to the physical presence of the exchanger or to its function in epithelial cells. Since kAE1 interacts with a number of cytosolic proteins^22^, it may act as a scaffolding protein that modulates tight junction properties. To test this possibility, we generated an mIMCD3 cell line that expresses the mutant protein kAE1 E681Q, which is unable to perform chloride/bicarbonate exchange^23,24^. As shown in Fig. 2A, upon incubation of mIMCD3 cells with doxycycline, the kAE1 E681Q mutant was expressed and migrated as two main bands similar to kAE1 WT protein, corresponding to a population of high mannose-carrying and complex-carrying kAE1 E681Q protein^15,18,20^. Although kAE1 E681Q mutant exhibited a higher percentage of high mannose-carrying population than kAE1 WT (47 ± 2% versus 9 ± 5%, respectively, n = 3), they were similarly localized in non-polarized cells or polarized cells where they both colocalized with the basolateral membrane marker β-catenin (Fig. 2B,C). Importantly, we confirmed that kAE1 E681Q mutant’s function was not significantly different from the control (mIMCD3 cells not expressing kAE1) but was significantly lower than the kAE1 WT function (Fig. 2D). We therefore confirmed that this mutant is functionally inactive although expressed at the basolateral membrane. We next performed Ussing chamber experiments and found that expression of the kAE1 E681Q mutant neither altered the TEER nor the absolute permeabilities to sodium or chloride in mIMCD3 monolayers **(**Fig. 3**)**. These results indicate that the effect of kAE1 protein on the tight junction permeability is dependent on its function rather than solely on its physical presence.

### kAE1 interacts with the tight junction protein claudin-4

**Figure 4:**
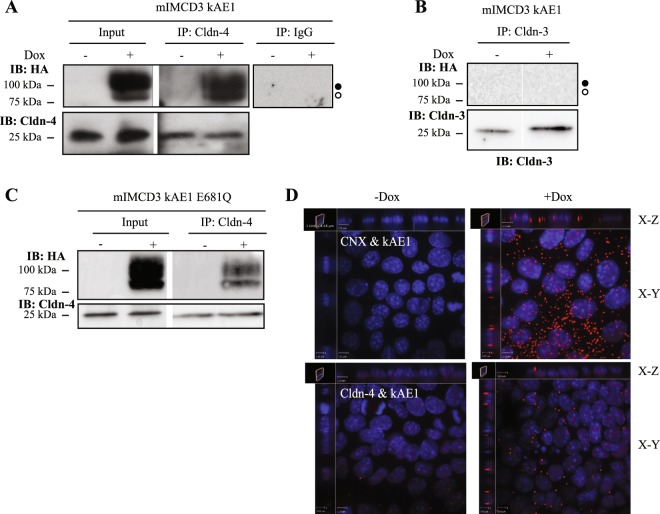
kAE1 protein is in close proximity and interacts with claudin-4. (**A**) Confluent mIMCD3 cells stably expressing kAE1 protein in an inducible manner were grown for 10 days in a 10 cm dish, lysed (Input) and claudin-4 was immunoprecipitated using a rabbit anti-claudin-4 antibody (IP lanes). As a control an irrelevant IgG antibody was used (right panel). Eluted proteins were separated on SDS-PAGE gel prior to immunoblotting with a mouse anti-claudin-4 (bottom blot) or mouse anti-HA (top blot) antibody. Dark circle corresponds to kAE1 carrying complex oligosaccharide, white circle indicates kAE1 carrying high mannose oligosaccharide. (**B**) A similar experiment was performed but immunoprecipitating claudin-3. No kAE1 co-immunoprecipitated with claudin-3 (top panel). (**C**) The claudin-4 immunoprecipitation was repeated using mIMCD3 kAE1 E681Q cells and shows that claudin-4 interacts with the mutant. (**D**) Proximity ligation assay was performed on mIMCD3 cells stably expressing kAE1 grown for 10 days on semi-permeable filters. CNX, corresponding to calnexin, was used as a positive control^15^, Cldn-4 corresponds to claudin-4. Cells were examined under a confocal microscope using a 63 X objective and sections (X-Z) or top views (X-Y) are shown. Red signal indicates that the 2 proteins labeled are within 30 to 40 nm of distance from each other. Nuclear staining is shown in blue as DAPI staining.

To determine by what mechanism kAE1 protein decreases TEER, we performed a membrane yeast two-hybrid assay in collaboration with Dr. Reinhart Reithmeier (University of Toronto, personal communication, February 2013) and found that kAE1 physically interacts with the tight junction protein claudin-4. Claudin-4 is expressed in murine principal cells, intercalated cells^25^, MDCK cells^26^ and mIMCD3 cells^27^ where it forms a paracellular pore to chloride ions. To confirm our finding, we performed a co-immunoprecipitation in mIMCD3 cells expressing kAE1 WT. As shown in Fig. 4A, endogenous claudin-4 co-immunoprecipitated with both high mannose- and complex-containing kAE1 protein (left and middle top panels), indicating that claudin-4 interacts with kAE1 proteins located both in the endoplasmic reticulum, and at the Golgi and beyond. Neither an irrelevant IgG (Fig. 4A, right top panel) nor a claudin-3 antibody (Fig. 4B) precipitated kAE1, indicating that the interaction between kAE1 and claudin-4 is specific. Interestingly, the inactive kAE1 E681Q mutant also co-immunoprecipitated with claudin-4 (Fig. 4C), indicating that the interaction between claudin-4 and kAE1 is not sufficient to regulate tight junction properties.

**Figure 5:**
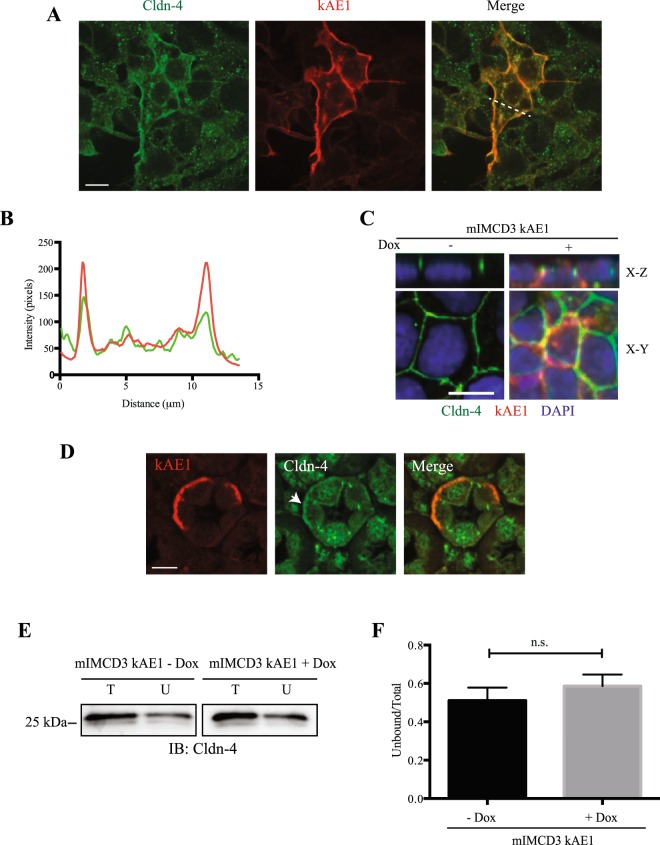
Claudin-4 colocalizes with kAE1 at the plasma membrane of mIMCD3 cells and in murine intercalated cells. (**A**), sub-confluent mIMCD3 cells expressing kAE1 were grown on glass coverslips, and incubated with doxycycline to induce kAE1 protein expression. Cells were then immunostained with an anti-HA antibody followed by Cy3 coupled secondary antibody (red) and anti-claudin-4 antibody (green). Bar = 20 μm. (**B**) Quantification of fluorescence intensities in the green and red channels along the dotted line shown in ***A*** (right panel), highlighting an enrichment of claudin-4 at the plasma membrane in non-polarized mIMCD3 cells. (**C**) mIMCD3 kAE1 cells were grown on semi-permeable filter for 10 days prior to immunostaining with anti-HA (red) and anti-claudin-4 (green) antibodies. Nuclei were stained with DAPI (blue). X-Y shows a middle section through the cells, X-Z shows a side view of the cells. Bar = 10 μm. (**D**) Mouse kidney sections were immunostained with anti-AE1 antibody (red) and anti-claudin-4 (green). Arrowhead indicates that in addition to its junctional localization, claudin-4 is detectable at the basal membrane of kAE1-positive intercalated cells of the renal collecting duct. (**E**) Cell surface biotinylation performed on polarized mIMCD3 kAE1 cells ± Dox. Total (T) and unbound (U) claudin-4 are shown. (**F**) quantification of the cell surface biotinylation results (U/T ratios), showing that there is no significant difference in the amount of cell surface claudin-4 upon kAE1 expression in polarized cells. Error bars correspond to means ± SEM, n = 7. n.s. indicates no significant (n.s.) difference with “mIMCD3 kAE1 – Dox” condition using un-paired t-test.

To further confirm our results, we asked whether claudin-4 and kAE1 co-localize by performing a proximity ligation assay (PLA, Fig. 4D) and an immunostaining on either mIMCD3 cells (Fig. 5A–C) or a mouse kidney section (Fig. 5D). The proximity ligation assay was performed on polarized mIMCD3 kAE1 cells using calnexin, a chaperone protein known to interact with newly synthesized kAE1^28^ as a positive control. Figure 4D depicts that in contrast to negative controls obtained in absence of doxycycline, induction of kAE1 protein expression resulted in red staining in cells stained with anti-calnexin antibody and more modestly in cells stained with the mouse anti-claudin-4 antibody. This finding confirms that claudin-4 and kAE1 proteins are within 30 to 40 nm distance to each other in polarized mIMCD3 cells. Interestingly, in cells stained for both kAE1 and claudin-4, X-Z sections showed staining at the upper lateral level of the polarized cells, supporting proximity of both proteins in the tight junction. Immunostaining of claudin-4 and kAE1 was performed on non-polarized and polarized mIMCD3 cells expressing kAE1 (Fig. 5A–C). In contrast with non-polarized mIMCD3 cells that did not express kAE1, claudin-4 appeared enriched at the plasma membrane in kAE1-expressing cells, as indicated by the line scan of both channels in Fig. 5B. In polarized mIMCD3 cells (Fig. 5C), however, there was no obvious relocation of claudin-4 to the basolateral membrane as claudin-4 was predominantly junctional, which may reflect that mIMCD3 cells do not polarize as extensively as other renal epithelial cell lines. Importantly, claudin-4 staining was discontinuous and irregular after polarization, possibly reflecting a reduced access of the epitope after polarization of the mIMCD3 cells. This finding supports that only a small fraction of kAE1 and claudin-4 proteins interact with each other, in agreement with the small amount of red dots seen in “Cldn-4 & kAE1” panel compared to “CNX & kAE1” panel in our PLA experiment (Fig. 4D**)**. To further assess whether the two proteins colocalize, we performed an immunostaining on mouse kidney sections. As shown in Fig. 5D, claudin-4 (green) was detectable at the tight junctions between cells, including between two kAE1-expressing cells, indicating that claudin-4 is expressed in type-A intercalated cells. Interestingly, claudin-4 was also detectable at the basal membrane of 31% of kAE1-expressing cells (arrowhead, 40 out of 129 cells observed). This finding supports that claudin-4 is not exclusively junctional but can be present at the basal membrane of some murine intercalated cells as well.

To assess whether kAE1 affected the abundance of claudin-4 at the tight junction/plasma membrane, we next quantified the amount of claudin-4 at the cell surface with or without kAE1 expression. Cell surface biotinylation was performed on mIMCD3 kAE1 cells grown to polarity, and total (T) versus non-biotinylated (U) fractions were compared. As shown in Fig. 5E,F, there was no significant difference between cell surface abundance of claudin-4 in the presence or absence of kAE1 protein, therefore supporting that kAE1 does not affect plasma membrane/junctional abundance of claudin-4. Together these results support that *(i)* at least a portion of kAE1 protein interacts with claudin-4 and *(ii)* claudin-4 surface abundance is not altered by kAE1 expression.

### The kAE1-induced decrease in TEER is mediated by claudin-4

**Figure 6:**
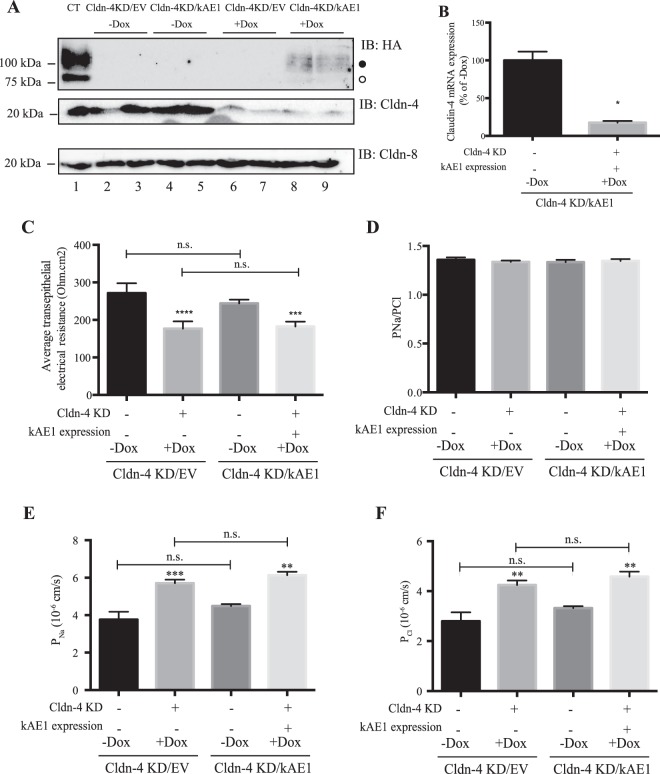
Claudin-4 knockdown reduces TEER and increases absolute permeability to both sodium and chloride. (**A**) mIMCD3 cells expressing kAE1, Cldn-4/EV or Cldn-4/kAE1 in an inducible manner were either incubated in presence or absence of doxycycline for 48 hours, prior to lysis and analysis of protein abundance by immunoblot. Mouse anti-HA antibody was used to detect kAE1 protein (upper panel), rabbit anti-claudin-4 (middle panel) or rabbit anti-claudin-8 (bottom panel) antibodies were used respectively to detect claudin-4 and claudin-8. The dark circle corresponds to kAE1 carrying complex oligosaccharide, and the white circle indicates kAE1 carrying high mannose oligosaccharide. Twenty micrograms of proteins were loaded in every lane. (**B**) Quantitative PCR analysis of claudin-4 mRNA abundance in Cldn-4 KD/kAE1 incubated in control conditions or with Doxycycline. The results are expressed as a percentage of “Cldn-4 KD/kAE1 – Dox” conditions and are normalized to the expression of actin. Error bars correspond to means ± SEM, n = 3. *P < 0.05 versus “Cldn-4 KD/kAE1 - Dox” condition using un-paired t-test. (**C**) Ussing chambers data showing an equivalent decrease in TEER in both Cldn-4 KD/EV and Cldn-4/kAE1 cells incubated with doxycycline. Error bars correspond to means ± SEM, n = 4. ***P < 0.001 versus Cldn-4 KD/kAE1 cells without doxycycline, ****P < 0.0001 versus Cldn-4 KD/EV cells without doxycycline. n.s. indicates non significant difference (One-way ANOVA). Although P_Na_ / P_Cl_ ratio was unchanged, (**D**), absolute permeability to both sodium (P_Na_) (**E**) or chloride (P_Cl_) (**F**) were increased upon Doxycycline incubation of Cldn-4/EV and Cldn-4/kAE1 cells. Error bars correspond to means ± SEM, n = 4, ***P < 0.001 versus Cldn-4 KD/EV cells without doxycycline, **P < 0.01 versus the same cell line without doxycycline.

To assess whether the interaction between kAE1 and claudin-4 was relevant to the decrease of TEER observed in mIMD3 cells upon kAE1 expression, we knocked down endogenous claudin-4 using small hairpin RNA (shRNA). To minimize compensatory up- or down-regulation of other tight junction proteins as previously shown with claudin-4 constitutive knockdown^29^, we aimed at preparing cell lines where claudin-4 knockdown was also inducible. However, we were not able to find a commercial shRNA system inducible with a drug other than doxycycline. Therefore, to assess the individual effect of claudin-4 knockdown over that of kAE1 expression, we generated two cell lines: a mIMCD3 cell line which upon doxycycline incubation expressed siRNA that resulted in the knockdown of endogenous claudin-4 (referred to here-after as Cldn-4 KD/EV), and a second mIMCD3 cell line in which doxycycline induced both claudin-4 knockdown and kAE1 expression (namely Cldn-4 KD/kAE1). Figure 6A shows an immunoblot confirming that after 48 hours of incubation of the cells with doxycycline, both kAE1 was expressed (lanes 8 & 9), albeit only to 20% of the control level (lane 1), and claudin-4 was knocked down by approximately 70% (lanes 6–9). Quantitative PCR also confirmed the knockdown of claudin-4 mRNA (Fig. 6B). Despite two separate attempts to generate these cells, we were unable to obtain higher kAE1 expression. As claudin-4 was reported to interact with claudin-8^30^, we verified whether claudin-4 knockdown affected claudin-8 abundance. The immunoblot in Fig. 6A shows that reducing claudin-4 abundance in these cells did not affect claudin-8 expression level.

**Figure 7:**
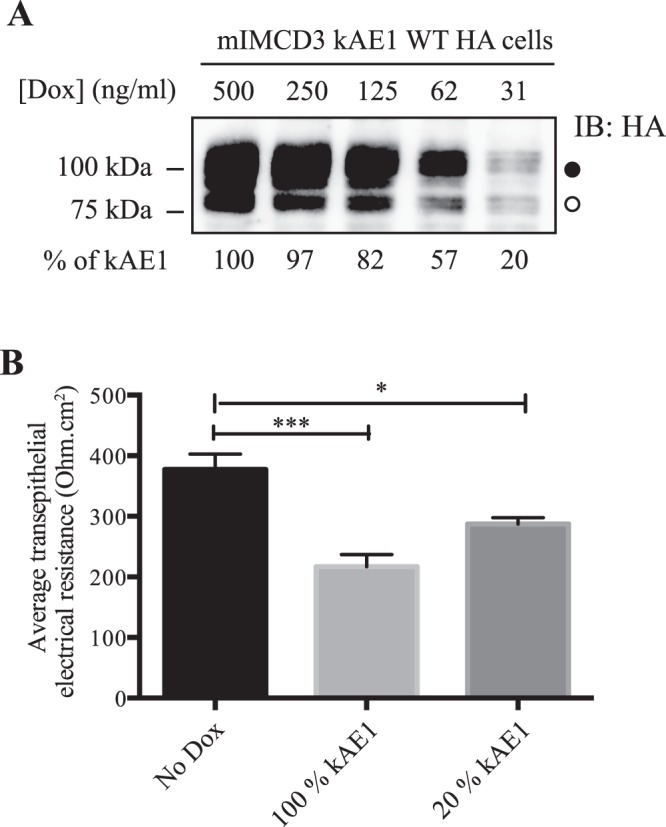
20% of kAE1 expression is enough to significantly alter tight junctions properties. (**A**) Immunoblot from mIMCD3 kAE1 cells incubated for 24 hours with various concentrations of doxycycline, and relative quantification of kAE1 abundance in these cells (bottom numbers). Incubation of the cells with 31 ng/ml of doxycycline was determined to induce 20% kAE1 expression compared to the routine 500 ng/ml doxycycline incubation. The dark circle corresponds to kAE1 carrying complex oligosaccharide, and the white circle indicates kAE1 carrying high mannose oligosaccharide. (**B**) Ussing chamber data showing that compared to cells that do not express kAE1 (No Dox), a 20% kAE1 expression results in significant decrease in TEER. Error bars correspond to means ± SEM, n = 4, ***P < 0.001 versus mIMCD3 kAE1 cells without doxycycline, *P < 0.05 versus mIMCD3 kAE1 cells without doxycycline.

We next examined the electrophysiological properties of these cell lines in Ussing chambers. In Cldn-4 KD/EV mIMCD3 cells, knock-down of claudin-4 was associated with a significant decrease in TEER (271 ± 37 ohm*cm^2^ in “– Dox” versus 177 ± 9 ohm*cm^2^, in “+Dox”, n = 4, Fig. 6C) consistent with a previous report^31^. In contrast to results from Fig. 1D, when claudin-4 was knocked down, kAE1 expression did not significantly affect the TEER (177 ± 9 ohm*cm^2^ in “Cldn-4/EV” versus 182 ± 6 ohm*cm^2^, in “Cldn-4/kAE1”, n = 6). This could be due to the low kAE1 expression in Cldn-4 KD/kAE1 cells (20% of the control cells, Fig. 6A). To test this hypothesis, we induced 20% of kAE1 expression and measured TEER in these conditions. Upon incubation of kAE1-expressing mIMCD3 cells with 31 ng/ml of doxycycline [which induced a 20% kAE1 expression compared to maximal expression (Fig. 7A)], the TEER significantly decreased to 287 ± 10 ohm*cm^2^ compared to 377 ± 25 ohm*cm^2^ in absence of Dox incubation (n = 4) (Fig. 7B). This indicates that a 20% kAE1 expression is enough to induce a 90 ohm*cm^2^ decrease in TEER. Given the absence of a decrease in TEER upon 20% kAE1 expression in claudin-4 knockdown cells, we interpret this result as kAE1 expression no longer reducing TEER in the absence of claudin-4. Together, these data support that the effect of kAE1 on tight junction properties is mediated through claudin-4.

### kAE1 WT expression acidifies the cytosolic pH, and alkalinize the pH and decreases chloride concentration of the extracellular basolateral medium

**Figure 8:**
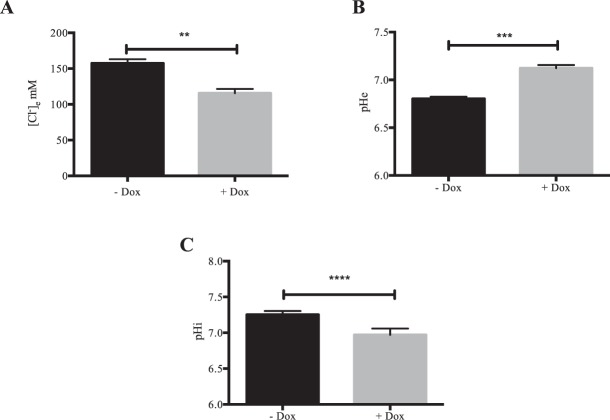
kAE1 expression alters cytosolic pH and chloride concentration and pH of the basolateral growth medium. Chloride concentration (**A**) and pH (**B**) of the basolateral growth medium were measured on mIMCD3 kAE1 cells grown for 8 days on semi-permeable filters and kept un-induced or induced with doxycycline for 24 hours. (**C**) Initial cytosolic pH was measured for the 20 seconds before switching from a chloride-containing to a chloride-free Ringer’s perfusion solution in mIMCD3 kAE1 cells kept un-induced or induced for 24 hours with doxycycline. Error bars correspond to means ± SEM, n = 3–7, **P < 0.01, ***P < 0.001 and ****P < 0.0001 versus mIMCD3 kAE1 cells without doxycycline.

To start deciphering the molecular mechanisms of kAE1 effect on tight junction properties and claudin-4, we addressed whether kAE1 expression in mIMCD3 cells alters cytosolic and/or extracellular ionic concentrations. After growing kAE1 mIMCD3 cells for 8 days on semi-permeable filters and a 24 hour-induction of kAE1 expression with doxycycline, we measured the basolateral growth medium chloride concentration and pH. As shown on Fig. 8, we observed a significant alkalization of the pH medium from 6.81 ± 0.01 in “–Dox” to 7.12 ± 0.02 in “+Dox” (n = 3) concomitant with a reduction of chloride concentration in the basolateral growth medium from 157 ± 6 mM in “– Dox” to 116 ± 6 mM in “+Dox” (n = 3) upon kAE1 expression, supporting that the activity of kAE1 WT alters the basolateral growth medium composition. Additionally, we measured the initial cytosolic pH in cells with or without incubation of doxycycline for 24 h and observed a significant acidification of the cytosolic pH in cells expressing kAE1 WT compared to the absence of kAE1 (Fig. 8C). Of note, the functionally inactive kAE1 E681Q mutant did not significantly change the cytosolic pH (data not shown). Together, these results support that the function of kAE1 WT affects cytosolic and extracellular pH and basolateral growth medium chloride concentration, which may alter claudin-4 function and thereby mediate the effect on tight junction properties.

## Discussion

In this manuscript we provide evidence that kAE1 expression and function alters tight junction permeability in renal epithelial cells. Our results therefore align with the effect of kAE1 expression in polarized MDCK I cells observed by Toye and colleagues^15^. To confirm their results and further investigate this effect, we generated a mIMCD3 cell line stably expressing kAE1 upon doxycycline induction, using the Clontech pLVX-TRE3G inducible expression system. We observed that upon incubation of the cells with doxycycline, kAE1 protein was synthesized, consisting of a high mannose-carrying and a complex-carrying population of proteins at steady state^15,18,20^, which predominantly localized to the basolateral membrane and was functional (Fig. 1). Using Ussing chambers, we confirmed that inducible expression of kAE1 protein reduced the TEER of renal mIMCD3 epithelial cells. This data indicates that the effect of kAE1 on TEER is not cell type specific as it has now been observed in two distinct cell lines, mIMCD3 (our data) and MDCK I cells (Toye and colleagues)^15^. Although the increased absolute transepithelial permeability to chloride might reflect kAE1-induced transcellular chloride permeability, kAE1 was not expected to increase transcellular sodium permeability as it acts as a chloride-bicarbonate exchanger. We thus propose that the absolute permeability to sodium (and possibly that to chloride) is increased via the paracellular pathway. Our results further support that the effect of kAE1 on TEER is mediated by the tight junction protein claudin-4.

A four transmembrane domain protein, claudin-4 is expressed in both intercalated and principal cells of the collecting duct^27,32,33^, as well as in the thin ascending limb of the loop of Henle^27,34^. This protein acts as an aldosterone-regulated and osmosensitive paracellular sodium barrier and chloride pore depending on the cell type and clone^35,36^. Claudin-4 contains a PDZ binding site in its cytosolic carboxyl terminal domain, which interacts with PDZ proteins such as MUPP1 or ZO-1 to maintain its location at the tight junction^31^. Aldosterone regulates claudin-4 abundance via threonine phosphorylation, which leads to a sharp decrease in TEER^36^. Importantly, claudin-4 physically interacts with claudin-8^30^, which promotes trafficking of the former to tight junctions. Although claudin-4 total knockout mice develop lethal hydronephrosis at one year of age^33^, principal cell-specific claudin-4 knockout leads to hypotension, hypochloremia and metabolic alkalosis caused by a severe renal wasting of chloride, without any alteration in claudin-8 abundance^27^, in agreement with our results (Fig. 6A). In contrast, claudin-8 knockout induced mistargeting and intracellular retention of claudin-4 protein^37^.

We found that endogenous claudin-4 knockdown in mIMCD3 cells results in a significant decrease in TEER, in agreement with a previous report in the same cell line^31^ (Fig. 6). However, our results differ from those of Gong and colleagues who observed that claudin-4 knockdown increased TEER^27,30^. This discrepancy might be either due to a clonal variation of the mIMCD3 cells used, or to the experimental protocol used for measuring TEER, as we did not incubate our cells with transcellular transporter inhibitors in order to preserve kAE1 function. Interestingly, in our claudin-4 KD mIMCD3 cells (Cldn-4 KD/EV), both absolute chloride and sodium permeabilities were increased (Fig. 6E,F), which may indicate that in our cell line, claudin-4 acts as a chloride and sodium barrier, or that it works in concert with another claudin providing a chloride barrier. Nevertheless, it is notable that either kAE1 expression (Fig. 1F,G) or claudin-4 KD has a similar effect on TEER, and absolute permeabilities to sodium and chloride.

To address whether the kAE1 effect on trans-epithelial properties was somehow related to claudin-4 while minimizing compensatory up-/down-regulations of other proteins, we tried to use two inducible systems to express kAE1 while knocking-down claudin-4. As our attempts to find non-doxycycline dependent inducible commercially available shRNA were unsuccessful, we used the Cldn-4 KD/EV cell line as a control for the Cldn-4 KD/kAE1 cells. Regardless kAE1 failed to significantly alter TEER in cells knocked down for claudin-4 (Fig. 6C), inferring that the effect of kAE1 on tight junctions is mediated by claudin-4.

kAE1 expression induced an increased absolute permeability to both chloride and sodium ions although claudin-4 is reported to only act as a chloride pore but not as a sodium pore^30^. This un-expected increase in sodium permeability may either indicate a disorganization of tight junctions upon kAE1 expression or a more specific effect on tight junction proteins. As claudin-4 physically interacts with claudin-8^30^, it is possible that alteration of claudin-4′s function results in secondary modification of (an)other claudin(s) function, possibly claudin-8. Although we did not detect a significant change in claudin-8 protein abundance in claudin-4 knockdown cells (Fig. 6A), it remains possible that kAE1 expression results in alteration of its location or phosphorylation state in these cells. Such an effect of the loss of one claudin on another claudin has been documented for claudin-16 and claudin-19, whose loss of activity causes familial hypomagnesemia with hypercalciuria and nephrocalcinosis (FHHNC)^38,39^. Thus, we propose that kAE1-induced decrease in epithelial tightness is rather due to a specific remodeling of cationic and anionic paracellular pores, which will need to be investigated in future studies.

Results from the proximity ligation assay support that both claudin-4 and kAE1 are within a 30 to 40 nm distance from each other (Fig. 4D). Interestingly, we observed a signal at the upper level of the lateral membrane, likely corresponding to tight junctions. A proximity ligation assay performed on non-polarized kAE1 expressing mIMCD3 cells also revealed an intracellular signal (data not shown), supporting that the interaction occurs before full differentiation and polarization of the epithelial cells. In agreement with this finding, co-immunoprecipitation of claudin-4 shows an interaction with both immature, high mannose-carrying kAE1 as well as mature, complex-carrying kAE1 (Fig. 4A). As immature kAE1 corresponds to kAE1 protein located in the endoplasmic reticulum (ER), this data is consistent with a physical interaction between the two proteins occurring as early as in the ER. Interestingly, in claudin-4 knockdown mIMCD3 cells, we were unable to detect kAE1 at an expression level similar to cells expressing claudin-4, despite two independent attempts to generate these cells (Fig. 6). Together with the interaction occurring early in the processing pathway, it is tempting to speculate that claudin-4 possibly plays a chaperone-like effect on kAE1 processing and possibly stability, a hypothesis that will also need to be further investigated.

kAE1 is predominantly a basolateral membrane protein while claudin-4 is primarily junctional in polarized cells. Based on the proximity ligation assay performed on polarized cells, a small portion of kAE1 may be located at the tight junction. Immunolocalization and cell surface biotinylation of claudin-4 in polarized control or kAE1-expressing mIMCD3 cells reveal no striking change in claudin-4 localization or plasma membrane abundance (Fig. 5C,E,F). Given the colocalization of kAE1 and claudin-4 in mouse kidney sections (Fig. 5D) and at the cell surface in non-polarized mIMCD3 cells (Fig. 5A), we were surprised by the lack of obvious colocalization after mIMCD3 cell polarization. This may be due to the poor polarization of these cells, which makes it difficult to assess subtle localization changes by confocal microscopy. Alternatively, it is possible that the claudin-4 epitope becomes less accessible to the antibody upon integration to tight junctions. Finally, these observations may suggest that these cells or their growth conditions do not truly model the *in vivo* environment of renal intercalated cells. The colocalization of claudin-4 and kAE1 at the plasma membrane in non-polarized kAE1-expressing mIMCD3 cells (Fig. 5A,B) and at the basal membrane of mouse kidney sections (arrowhead, Fig. 5D) raises the possibility that expression of kAE1 may have relocated a portion of claudin-4 from the tight junction to the basolateral membrane, thereby altering tight junction properties, without changing its overall cell surface abundance (Fig. 5E,F). The relocation of claudins from tight junction to either the cytosol or the lateral membrane has been previously described in intestinal and pancreatic epithelial cells^40–42^, highlighting that their localization is highly dynamic. A kAE1-dependent partial relocation of claudin-4 may support a fast and possibly reversible modulation of tight junction properties in response to acute homeostatic changes. Alternatively, the alteration of cytosolic concentration of protons (and therefore likely of chloride) in the vicinity of claudin-4 due to kAE1 activity (Fig. 8) may result in a change in claudin-4 phosphorylation status and therefore activity^36^. This latter change may allow a fast and reversible change in tight junction properties. This second hypothesis is in line with the loss of effect on tight junction properties upon expression of the functionally dead kAE1 E681Q mutant (Fig. 3).

At this time, the exact interaction site on kAE1 and claudin-4 primary structure is unknown. Our preliminary data using a peptide spot assay consisting of the amino acid sequence of kAE1 and overlaid with claudin-4-containing lysates support that claudin-4 interacts with the third and fourth extracellular loop as well as with the cytosolic N-terminus of kAE1 (data not shown). kAE1 possesses a carboxyl-terminal PDZ binding site^9^, which could possibly be involved in the interaction with un-identified scaffolding protein(s) enriched at the tight junctions, and retention of this sub-population thereby allowing a physical interaction with claudin-4.

Our experiments showed that rather than kAE1 playing a scaffolding effect on tight junctions in a similar way as erythroid AE1 links cytoskeleton to plasma membrane in red blood cells^43^, its activity appears to be crucial for its effect on tight junctions. Importantly, Toye and colleagues reported that the functional dRTA mutant kAE1 R901X which was mis-trafficked to the apical membrane in MDCK I cells, did not alter the epithelium tightness^15^. This finding indicates that the function of an active chloride/bicarbonate transporter in another location than either the basolateral membrane or tight junction is not sufficient to affect renal epithelial tightness. Therefore, our data together with Toye’s results suggest that the regulation of tight junction properties by kAE1 is not only dependent on the function of the bicarbonate exchanger but possibly also on the close vicinity with claudin-4, although we don’t have additional experimental evidence other than Toye’s results supporting the latter at this point. We speculate that at interaction sites between claudin-4 and kAE1 at the level of tight junctions, kAE1’s function generates a microenvironment where either or both chloride and bicarbonate local concentrations vary acutely, thus resulting in rapid alterations of claudin-4 function. Importantly, claudin-4 interacts with claudin-8 which itself regulates paracellular permeability to acidic and basic ions^44^, therefore it is also possible that the claudin-4/kAE1 interaction alters paracellular flux of hydroxyl ions or protons, and plays a role in acid-base homeostasis. Finally, another alternative is that kAE1 expression may relocate a portion of claudin-4 away from tight junctions in certain conditions, thereby affecting tight junction properties. Interestingly, a study from Sebastian and colleagues reported that RTA patients waste renal sodium and chloride even with sustained oral administration of potassium bicarbonate^12^. Upon low dietary intake of sodium, these patients were unable to conserve sodium and chloride. We speculate that our findings highlighting a role for claudin-4/kAE1 interaction in regulating TEER and ion permeability could possibly account for the reported loss of urinary sodium and chloride in dRTA patients^12^. We propose that in dRTA patients where kAE1 function is abnormal due to a mutation in the SLC4A1 gene, the functional interaction between kAE1 and claudin-4 is altered, thus resulting in a tighter collecting duct epithelium preventing sodium and chloride reabsorption^45^ and thus loss of urinary ions. This loss of urinary electrolytes would therefore not be improved by sustained correction of metabolic acidosis as it would be caused by a defective ability of kAE1 to reduce the trans-epithelial resistance, a hypothesis that will need to be tested in further studies.

## Materials and Methods

### Plasmid constructs and antibodies

The pLVX TRE3G kAE1 construct was generated by shuttling human kAE1 cDNA carrying an external hemaglutinin (HA) epitope in position 557 into pLVX-TRE3G plasmid (Clontech) using MluI restriction sites in 3′ and 5′ of the open reading frame. The insertion of the HA epitope did not alter kAE1 localization or function^18^. The sequence of the full construct was then verified using automated sequencing (The Applied Genomics, Edmonton, AB). This construct has been designated as “kAE1” throughout the manuscript. The kAE1 E681Q mutant was generated using the Q5 site-directed mutagenesis kit (NEB) and mutagenesis primers with the following sequences: forward: CATATTCCTGCAGTCTCAGATCAC; reverse: AGGATGAAGACCAGCAGAG. Three different inducible claudin-4 shRNA-containing lentiviruses were purchased from Dharmacon (Dharmacon SMARTvector Inducible Lentiviral shRNAs, GE Healthcare) that enclosed siRNA hairpin oligonucleotides with one of the following sequences: CAAAGTTACTAGCCCGTAG (shRNA#1), GGACCGCTCACAACGTCAT (shRNA#2) and CCGGAGCCGTGTTCATCGT (shRNA#3). The siRNAs were downstream of the mouse CMV promoter, and were followed by a Turbo RFP reporter to track transduction and expression upon doxycycline induction. Polyclonal rabbit antibodies detecting murine claudin-4 or claudin-3 used for immunoblot or immunoprecipitation was purchased from InVitrogen (Cat # 36-4800 and 34-1700, respectively), monoclonal mouse anti-claudin-4 antibody coupled to AlexaFluor488 (3E2C1) was used for immunofluorescence and was obtained from Thermoscientific (Cat # 329488), polyclonal rabbit anti-claudin-8 antibody was purchased from InVitrogen (Cat # 40-2600), polyclonal rabbit anti-calnexin is a kind gift from Dr. David Williams (U of Toronto), polyclonal rabbit anti-β-Catenin antibody (Cat # C2206) was obtained from Sigma-Aldrich, USA. Monoclonal mouse anti-HA antibody was obtained from Covance (Cat # MMS-101R) and subsequently BioLegend (Cat # 901513).

### Cell Culture

#### IMCD3 cells inducibly expressing kAE1 WT-HA, kAE1 E681Q-HA or empty vector (EV)

70% confluent Lenti-X 293 T cells (Clontech) were transfected with either 7 μg of pLVX-Tet3G regulator plasmid, pLVX-TRE3G kAE1 WT-HA (later referred as kAE1), pLVX-TRE3G kAE1 E681Q-HA, or empty pLVX-TRE3G (pLVX-TRE3G EV) response plasmids using Lenti-X HTX Packaging mix and Xfect Transfection Reagent (Clontech), following the manufacturer’s instructions. Cells were incubated at 37 °C in serum-free OptiMEM medium (Gibco) for 48 hours. Supernatants containing the lentivirus were collected and filtered through 0.45 μm filters. 70% confluent mIMCD3 (ATCC, CRL-2123) were then co-infected with lentiviruses containing both regulator (pLVX-Tet3G) and response (pLVX-TRE3G kAE1 WT-HA, pLVX-TRE3G kAE1 E681Q-HA, or pLVX-TRE3G EV) plasmids and 4 μg/ml of polybrene (Sigma-Aldrich, USA) for 48 hours. The growth medium was then complemented with 4 μg/ml Puromycin and 2 mg/ml G418 to select infected cells. Expression of kAE1 proteins was optimally detected after incubation with 0.5 μg/ml of doxycycline for 24 hours. Upon doxycycline induction, these cells expressed similar amounts of kAE1 even after multiple passages.

#### Lentiviruses shRNA against claudin-4, EV or kAE1 expressing cells

To generate claudin-4 knocked down (Cldn-4 KD) cells, 3 different claudin-4 shRNA were used. 10^5^ mIMCD3 cells were plated on a 6 well plate and transduced with each shRNA-containing lentiviruses (titer ranging from 4.5 to 6.5 10^7^ TU/ml) to reach a MOI of 6, in the presence of 4 μg/ml of polybrene in serum-free OptiMEM medium for 48 hours at 37 °C. Transduction medium was replaced by an antibiotic free medium for 24 hours followed by incubation with selection medium containing 4 μg/ml of Puromycin. Claudin-4 shRNA expression was optimally induced with 5 μg/ml Doxycycline for 48 hours. To generate the mIMCD3 cell line with both inducible knockdown of claudin-4 and expression of kAE1 protein or empty vector, we co-infected Cldn-4 KD cells with lentiviruses that either contained regulator (pLVX-Tet3G) and response (pLVX-TRE3G kAE1 WT-HA or pLVX-TRE3G EV) plasmids for 48 hours at 37 °C. We therefore generated 2 cell lines: “Cldn-4 KD/EV”, which upon induction with doxycycline are knocked down for claudin-4, and “Cldn-4 KD/kAE1”, which upon doxycycline induction express kAE1 and are knocked down for claudin-4. The cells were subsequently grown in a selection medium containing 4 μg/ml Puromycin and 2 mg/ml G418. Expression of kAE1 proteins and claudin-4 knockdown were induced using 5 μg/ml of Doxycycline for 48 hours.

### Functional Assay

70% confluent IMCD3 cells expressing kAE1 WT-HA were grown on 11 × 7.5 mm glass coverslips and treated as previously described^46^. Briefly, after three washes of the coverslips with serum-free OptiMEM medium (Gibco), the cells were incubated with 10 μM BCECF-AM (Sigma-Aldrich, USA) for up to 1 hour at 37 °C. Coverslips were then placed in fluorescence cuvettes at room temperature and the cells were perfused with Ringer’s buffer (5 mM glucose, 5 mM potassium gluconate, 1 mM calcium gluconate, 1 mM magnesium sulphate, 10 mM HEPES, 2.5 mM NaH_2_PO_4_, 25 mM NaHCO_3_, pH 7.35–7.45) containing 140 mM chloride. Intracellular alkalinization was then induced by replacing the perfusing solution with a chloride free Ringer’s buffer containing 140 mM gluconate. Finally, we calibrated the BCECF-AM intracellular fluorescence by perfusing the cells with buffers at pH 6.5, 7.5, or 7.0 supplemented with 100 μM nigericin sodium salt (Sigma-Aldrich, USA). A Photon Technologies International (PTI) (London, Ontario, Canada) fluorescence spectrometer was used to read the fluorescence emissions generated by the samples. Excitation wavelengths of 440 and 490 nm and emission wavelength of 510 nm were used. The transport rates were calculated using linear regression of the initial rate of intracellular alkalinization (first 60 seconds), normalized to pH calibration measurements. All measurements were done using Felix software.

### Immunoblotting and immunoprecipitation

Confluent or sub-confluent mIMCD3 cells expressing kAE1 WT-HA were lysed in RIPA lysis buffer (0.3 M NaCl, 20 mM Tris/HCl pH 7.5, 2 mM EDTA, 2% Deoxycholate, 2% Triton X-100, 0.2% SDS, pH 7.4) and protease inhibitors (1 μg/ml aprotinin, 2 μg/ml leupeptin, 1 μg/ml pepstatin A, and 100 μg/ml PMSF), and the total protein concentration measured using a BCA assay (Pierce, Rockford, IL, USA). 20 μg of proteins were loaded on a SDS-PAGE gel and kAE1 proteins in the lysates were detected by immunoblotting with mouse anti-HA or rabbit anti-claudin-4 antibodies overnight at 4 °C, followed by secondary antibodies coupled to horseradish peroxidase (HRP) for 1 hour at room temperature. Enhanced chemiluminescence (ECL prime western blotting detection reagent from GE Healthcare, Wauwatosa, WI, USA) and a BioRad Imager were used to detect proteins. Relative band intensities were determined using ImageLab software (BioRad). For immunoprecipitations, the lysate was incubated with 3 μl of rabbit anti-claudin-4 or anti-claudin-3 antibody for 2 hours on a rocker at 4 °C, followed by 30 μl of protein G-coupled sepharose beads (GE Healthcare) for an additional hour at 4 °C. Eluted proteins were separated by SDS-PAGE and claudin-4, claudin-3 or kAE1 proteins immunoblotted using either a mouse anti-claudin 4, mouse anti-claudin-3 or a mouse anti-HA antibody, respectively.

### Ussing Chambers experiments

Confluent mIMCD3 cells stably expressing kAE1 WT-HA in an inducible manner were grown on 0.45 μm semi-permeable Transwell filters (Costar, Cat # 07200225) for 10 to 30 days. 24 hours prior to the beginning of the experiment, kAE1 protein expression was induced by incubation of the cells with 0.5 μg/ml of doxycycline. The filters were then mounted in Ussing chambers and bathed at 37 °C in solution A containing: 145 mM NaCl, 1 mM CaCl_2_, 1 mM MgCl_2_, 10 mM glucose, and 10 mM HEPES, pH 7.4. The current was first clamped using a DVC 1000 I/V clamp (World Precision Instruments, Sarasota, FL) and electrodes filled with 3 M KCl. Data were recorded as a trace using PowerLab (ADInstruments, Colorado Springs, CO) and Chart 4.0 software. 90 μA pulses were applied across the epithelial monolayer and a dilution potential was induced by replacing solution A bathing the basolateral membrane with an iso-osmotic solution containing 80 mM NaCl (Solution B: 80 mM NaCl, 130 mM mannitol, 1 mM CaCl_2_, 1 mM MgCl_2_, 10 mM glucose, and 10 mM HEPES, pH 7.4) allowing us to calculate transepithelial electrical resistance (TEER), absolute permeabilities of the epithelium to sodium and chloride and ion permeability ratios using the Goldman-Hodgkin Katz and Kimizuka Koketsu equations, as described previously^29,47^.

### Proximity Ligation Assay

Confluent mouse IMCD3 cells expressing kAE1 WT-HA proteins were seeded on 12 mm glass cover slips and incubated with or without 0.5 µg/ml Doxycycline for 48 hours before the experiment day. The assay was performed according to the manufacturer’s instructions (Olink Bioscience, Sweden). Briefly, the cells were washed with 1 X PBS, fixed with 4% PFA, quenched with 50 mM NH_4_Cl, permeabilized with 0.1% Triton-X 100 and washed twice with buffer A (10 mM Tris, 150 mM NaCl, 0.05% Tween 20, pH 7.4; provided by the company, Olink Bioscience, Sweden). In a pre-heated humidified chamber, the cells were then blocked with 40 µl of the blocking solution (provided by the company; Olink Bioscience, Sweden) at 37 °C, prior to addition of 40 μl mouse anti HA and rabbit anti claudin-4 antibodies (1/500 in provided antibody diluent) for 1 hour at 37 °C. After washes, the two minus anti-mouse and the plus anti-rabbit PLA probes were added to each sample for 1 hour at 37 °C in the humidity chamber, followed by the ligation-ligase mixture for 30 minutes and finally with the amplification-polymerase mixture in the humidity chamber for 100 minutes. After 3 washes with 0.01 X of provided buffer B supplemented with DAPI, the coverslips were mounted on glass slides. Samples were examined using a 60 X oil objective with a WaveFX confocal microscope (Quorum Technologies, Canada).

### Immunohistochemistry

PBS/heparin-perfused 129S6/SvEvTac mouse kidneys were paraffin-embedded and sectioned every 2 μm prior to formalin-fixation as previously described^48^. Sections were then rehydrated using Tissue-Clear (Tissue-Tek, Dakura sections) and subsequently incubated in graded ethanol solutions into water and next submitted to heat induced antigen retrieval with a TEG solution (10 mM Tris, 0.5 mM EGTA, pH = 9.0). Sections were next incubated with 0.6% H_2_O_2_ and 50 mM NH_4_Cl in PBS to block endogenous peroxidases enzymes and free aldehyde groups. Sections were incubated with mouse anti-AE1 antibody (kind gift from Dr. Sebastian Frische) in 0.1% Triton X-100 in PBS at 4 °C followed by anti-mouse Cy3 secondary antibody and mouse anti-claudin-4 antibody coupled to Alexa488 prior to mounting with Aquamount. The kidney sections were observed using an Olympus IX81 microscope equipped with a Nipkow spinning-disk optimized by Quorum Technologies (Guelph, ON, Canada) and a 63 X oil objective.

### Immunofluorescence

Sub-confluent or polarized mouse IMCD3 cells expressing kAE1 WT-HA proteins were incubated with or without 0.5 µg/ml Doxycycline for 24 hours prior to fixation with 4% paraformaldehyde (Canemco Supplies) in PBS followed by incubation with 100 mM glycine in PBS (pH 8.5) to quench non-specific fluorescence. After cell permeabilization with 0.2% Triton X-100 in PBS and blocking with 1% BSA, the kAE1 proteins were detected with a mouse anti-HA primary antibody (Covance) followed by an anti-mouse antibody coupled to Cy3 (Jackson Immunoresearch Laboratories), while endogenous claudin-4 was detected with a mouse anti-claudin-4 antibody coupled to Alexa488. Nuclei were stained with DAPI. Coverslips were finally mounted onto glass slides using DAKO mounting solution and examined using an Olympus IX81 microscope equipped with a Nipkow spinning-disk optimized by Quorum Technologies (Guelph, ON, Canada) and a 63 X oil objective.

### Cell Surface Biotinylation

Polarized mouse IMCD3 cells expressing kAE1 WT-HA proteins were incubated with or without 0.5 µg/ml Doxycycline for 24 hours prior to two incubations with EZ-Link Sulfo-NHS-SS-Biotin reagent (1 mg/ml) (Pierce) at 4 °C for 15 min in borate buffer (10 mM Boric acid, 145 mM NaCl, 7.2 mM KCl, 1.8 mM CaCl_2_, pH 9). The excess of biotin was quenched 3 times (10 minutes each) with 100 mM glycine in PBS. The cells were lysed in 300 μl RIPA lysis buffer (10 mM Tris-HCl, 150 mM NaCl, 1 mM EDTA, 1% Triton X-100, 0.1% SDS, 1% deoxycholate, 1 μg/ml aprotinin, 2 μg/ml leupeptin, 1 μg/ml pepstatin A, and 100 μg/ml PMSF, pH 7.5). An aliquot (30 μl) of the lysate was kept as total lysate (T) and the remaining was incubated with streptavidin agarose resin (100 μl, Thermo Scientific) for 1 hour at 4 °C. An aliquot (30 μl) of unbound proteins (U) was saved. The same volume (30 μl) of “T” and “U” fractions were loaded on SDS-PAGE gel and samples were immunoblotted with a mouse anti-claudin-4 antibody. Relative band intensities were determined using the Image Lab software (BioRad). The results are expressed as unbound/total (U/T) ratios.

### Real-time quantitative PCR

Total mRNA was isolated from Cldn-4 KD/kAE1 mIMCD3 cells incubated with doxycycline for 48 hours or kept in control conditions, using EZ-10 DNAaway RNA Mini-Preps Kit, (BIOBasic Canada Inc, ON, Canada). DNAse I treatment was next performed, followed by reverse transcription of 5 µg RNA using SuperScript II transcriptase and random primers obtained from IDT (Integrated DNA Technologies, San Diego, CA). The cDNA was next used to determine claudin-4 and the internal control actin mRNA levels using primers from Thermo Fisher/ABI (claudin-4 primers and probe: Cat # Mm00515514-s1; actin primers and probe: Cat # Mm01205647-g1). Expression levels were determined by qPCR on an ABI Prism 7900 HT sequence detection System (Applied Biosystem, Foster City, CA).

### Measurements of growth medium chloride and pH

Initial cytosolic pH was measured for the last 20 seconds before switching perfusion with chloride-free Ringer’s buffer containing 140 mM gluconate as described in the section “Functional Assay”. Extracellular pH was measured with a pH microelectrode (Orion^TM^ PerpHect^TM^ ROSS^TM^ combination pH microelectrode, ThermoFisher) from fresh basolateral growth medium from kAE1 mIMCD3 cells grown on semi-permeable filters for 8 days and kept un-induced or induced for 24 hours with doxyxycline. Chloride concentration in the basolateral growth medium was measured on medium diluted 1:100 in double distilled water using Ion Chromatography (Dionex Aquion Ion Chromatography system Anion (Thermofisher), with Dionex Combined Five Anion Standards).

### Statistical analysis

All experiments were independently repeated a minimum of three times. Results are expressed as mean values ± standard error of the mean (SEM) and “n” indicates the number of experimental repeats. Statistical comparisons were either made using unpaired Student t-test or one-way ANOVA where appropriate, *P* < *0*.*05* was considered significant.

### Summary statement

In collecting duct cells, kAE1 alters tight junction properties via an effect dependent on both the function of the exchanger and the presence of claudin-4. The two proteins physically interact.

## Supplementary information

Supplementary Information

## References

[1] Arakawa T (2015). Crystal structure of the anion exchanger domain of human erythrocyte band 3. Science (80-.)..

[2] Cordat E, Reithmeier RAFA (2014). Structure, function, and trafficking of SLC4 and SLC26 anion transporters. Curr Top Membr.

[3] Vince JW, Reithmeier RA (1998). Carbonic anhydrase II binds to the carboxyl terminus of human band 3, the erythrocyte C1-/HCO3- exchanger. J Biol Chem.

[4] Almomani, E. Y., Touret, N. & Cordat, E. Adaptor protein 1 B mu subunit does not contribute to the recycling of kAE1 protein in polarized renal epithelial cells. *Mol*. *Membr*. *Biol*., 10.1080/09687688.2018.1451662 (2018). 10.1080/09687688.2018.1451662 29651904

[5] Almomani EY (2012). Adaptor protein 1 complexes regulate intracellular trafficking of the kidney anion exchanger 1 in epithelial cells. Am J Physiol Cell Physiol.

[6] Sawasdee N (2010). Human kidney anion exchanger 1 interacts with adaptor-related protein complex 1 mu1A (AP-1 mu1A). Biochem Biophys Res Commun.

[7] Su Y (2011). Glyceraldehyde 3-phosphate dehydrogenase is required for band 3 (anion exchanger 1) membrane residency in the mammalian kidney. Am J Physiol Ren. Physiol.

[8] Sorrell, S. L., Golder, Z. J., Johnstone, D. B. & Karet Frankl, F. E. Renal peroxiredoxin 6 interacts with anion exchanger 1 and plays a novel role in pH homeostasis. *Kidney Int*, 10.1038/ki.2015.277 (2015). 10.1038/ki.2015.277 PMC4705439 26398495

[9] Cordat E, Li J, Reithmeier RA (2003). Carboxyl-terminal truncations of human anion exchanger impair its trafficking to the plasma membrane. Traffic.

[10] Su Y (2017). PDLIM5 links kidney anion exchanger 1 (kAE1) to ILK and is required for membrane targeting of kAE1. Sci. Rep..

[11] Pereira PCB, Miranda DM, Oliveira EA, Silva ACSE (2009). Molecular pathophysiology of renal tubular acidosis. Curr. Genomics.

[12] Sebastian A, McSherry E, Morris RC (1976). Impaired renal conservation of sodium and chloride during sustained correction of systemic acidosis in patients with type 1, classic renal tubular acidosis. J Clin Invest.

[13] Quilty JAA, Cordat E, Reithmeier RAFA (2002). Impaired trafficking of human kidney anion exchanger (kAE1) caused by hetero-oligomer formation with a truncated mutant associated with distal renal tubular acidosis. Biochem J.

[14] Ungsupravate D (2010). Impaired trafficking and intracellular retention of mutant kidney anion exchanger 1 proteins (G701D and A858D) associated with distal renal tubular acidosis. Mol Membr Biol.

[15] Toye AM, Banting G, Tanner MJ (2004). Regions of human kidney anion exchanger 1 (kAE1) required for basolateral targeting of kAE1 in polarised kidney cells: mis-targeting explains dominant renal tubular acidosis (dRTA). J Cell Sci.

[16] Rungroj N (2004). A novel missense mutation in AE1 causing autosomal dominant distal renal tubular acidosis retains normal transport function but is mistargeted in polarized epithelial cells. J Biol Chem.

[17] Kittanakom S, Cordat E, Akkarapatumwong V, Yenchitsomanus PT, Reithmeier RA (2004). Trafficking defects of a novel autosomal recessive distal renal tubular acidosis mutant (S773P) of the human kidney anion exchanger (kAE1). J Biol Chem.

[18] Cordat E (2006). Dominant and recessive distal renal tubular acidosis mutations of kidney anion exchanger 1 induce distinct trafficking defects in MDCK cells. Traffic.

[19] Vichot AA (2017). Loss of kAE1 expression in collecting ducts of end-stage kidneys from a family with SLC4A1 G609R-associated distal renal tubular acidosis. Clin. Kidney J..

[20] Mumtaz R (2017). Intercalated Cell Depletion and Vacuolar H-ATPase Mistargeting in an Ae1 R607H Knockin Model. J. Am. Soc. Nephrol..

[21] Chu C (2010). Band 3 Edmonton I, a novel mutant of the anion exchanger 1 causing spherocytosis and distal renal tubular acidosis. Biochem J.

[22] Cordat E, Casey JRR (2009). Bicarbonate transport in cell physiology and disease. Biochem J.

[23] Jennings ML, Smith JS (1992). Anion-proton cotransport through the human red blood cell band 3 protein. Role of glutamate 681. J Biol Chem.

[24] Chernova MN (1997). Electrogenic sulfate/chloride exchange in Xenopus oocytes mediated by murine AE1 E699Q. J Gen Physiol.

[25] Chen L (2017). Transcriptomes of major renal collecting duct cell types in mouse identified by single-cell RNA-seq. Proc. Natl. Acad. Sci. USA.

[26] Sonoda N (1999). Clostridium perfringens enterotoxin fragment removes specific claudins from tight junction strands: Evidence for direct involvement of claudins in tight junction barrier. J. Cell Biol..

[27] Gong Y (2014). The Cap1-claudin-4 regulatory pathway is important for renal chloride reabsorption and blood pressure regulation. Proc Natl Acad Sci USA.

[28] Popov M, Reithmeier RA (1999). Calnexin interaction with N-glycosylation mutants of a polytopic membrane glycoprotein, the human erythrocyte anion exchanger 1 (band 3). J Biol Chem.

[29] Borovac J (2012). Claudin-4 forms a paracellular barrier, revealing the interdependence of claudin expression in the loose epithelial cell culture model opossum kidney cells. Am J Physiol Cell Physiol.

[30] Hou J, Renigunta A, Yang J, Waldegger S (2010). Claudin-4 forms paracellular chloride channel in the kidney and requires claudin-8 for tight junction localization. Proc Natl Acad Sci USA.

[31] Lanaspa MA, Andres-Hernando A, Rivard CJ, Dai Y, Berl T (2008). Hypertonic stress increases claudin-4 expression and tight junction integrity in association with MUPP1 in IMCD3cells. Proc. Natl. Acad. Sci..

[32] Kiuchi-Saishin Y (2002). Differential expression patterns of claudins, tight junction membrane proteins, in mouse nephron segments. J Am Soc Nephrol.

[33] Fujita H, Hamazaki Y, Noda Y, Oshima M, Minato N (2012). Claudin-4 deficiency results in urothelial hyperplasia and lethal hydronephrosis. PLoS One.

[34] Günzel D, Yu ASL (2013). Claudins and the modulation of tight junction permeability. Physiol. Rev..

[35] Conner SD, Schmid SL (2003). Regulated portals of entry into the cell. Nature.

[36] Le Moellic C (2005). Aldosterone and tight junctions: modulation of claudin-4 phosphorylation in renal collecting duct cells. Am J Physiol Cell Physiol.

[37] Gong Y (2015). KLHL3 regulates paracellular chloride transport in the kidney by ubiquitination of claudin-8. Proc. Natl. Acad. Sci. USA.

[38] Hou J (2008). Claudin-16 and claudin-19 interact and form a cation-selective tight junction complex. J. Clin. Invest..

[39] Hou J (2009). Claudin-16 and claudin-19 interaction is required for their assembly into tight junctions and for renal reabsorption of magnesium. Proc. Natl. Acad. Sci..

[40] Inai T, Sengoku A, Hirose E, Iida H, Shibata Y (2007). Claudin-7 Expressed on Lateral Membrane of Rat Epididymal Epithelium does not Form Aberrant Tight Junction Strands. Anat. Rec. Adv. Integr. Anat. Evol. Biol..

[41] Westmoreland JJ (2012). Dynamic distribution of claudin proteins in pancreatic epithelia undergoing morphogenesis or neoplastic transformation. Dev. Dyn..

[42] Pasternak JA, Kent-Dennis C, Van Kessel AG, Wilson HL (2015). Claudin-4 undergoes age-dependent change in cellular localization on pig jejunal villous epithelial cells, independent of bacterial colonization. Mediators Inflamm..

[43] Satchwell TJ, Hawley BR, Bell AJ, Ribeiro ML, Toye AM (2015). The cytoskeletal binding domain of band 3 is required for multiprotein complex formation and retention during erythropoiesis. Haematologica.

[44] Angelow S, Kim KJ, Yu AS (2006). Claudin-8 modulates paracellular permeability to acidic and basic ions in MDCK II cells. J Physiol.

[45] Gueutin V (2013). Renal beta-intercalated cells maintain body fluid and electrolyte balance. J Clin Invest.

[46] Chu CY, King JC, Berrini M, Alexander RT, Cordat E (2013). Functional rescue of a kidney anion exchanger 1 trafficking mutant in renal epithelial cells. PLoS One.

[47] Kimizuka H, Koketsu K (1964). Ion transport through cell membrane. J. Theor. Biol..

[48] Alexander RT (2015). Ultrastructural and immunohistochemical localization of plasma membrane Ca ^2+^ -ATPase 4 in Ca ^2+^ -transporting epithelia. Am. J. Physiol. Physiol..

