# Supplementary material for: The kidney anion exchanger 1 affects tight junction properties via claudin-4
Source: Sci Rep. 2019 Feb 28;9:3099. doi: 10.1038/s41598-019-39430-9 (PMC6395713; doi:10.1038/s41598-019-39430-9)
Supplement: Supplementary file 1 — Supplementary Information [file 41598_2019_39430_MOESM1_ESM.pdf]

The kidney anion exchanger 1 affects tight junction properties via claudin-4

Rawad Lashhab*, Alina C. Rumley*, Denis Arutyunov*, Midhat Rizvi*, Charlotte You*, Henrik Dimke[$], Nicolas Touret[&], Richard Zimmermann[#], Martin Jung[#], Xing-Zhen Chen*, Todd Alexander* & Emmanuelle Cordat*[¶]

Supplementary Information

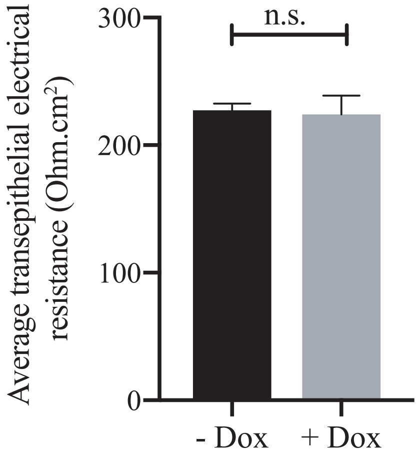

Average transepithelial electrical resistance (Ohm.cm2)
300
200
100
0
n.s.
- Dox
+ Dox

Supplementary Figure. Doxycycline incubation does not alter transepithelial electrical resistance. Non-infected polarized mIMCD3 cells were kept un-induced or induced for 24 hours with doxycycline and the Ussing chamber experiments were performed as described in the Methods section. Error bars correspond to means ± SEM, n=3-4, there was no significant (n.s.) difference between - Dox and + Dox conditions.
